# Expression of angiotensinogen and receptors for angiotensin and prorenin in the monkey and human substantia nigra: an intracellular renin–angiotensin system in the nigra

**DOI:** 10.1007/s00429-012-0402-9

**Published:** 2012-03-11

**Authors:** Pablo Garrido-Gil, Rita Valenzuela, Begoña Villar-Cheda, Jose L. Lanciego, Jose L. Labandeira-Garcia

**Affiliations:** 1 Laboratory of Neuroanatomy and Experimental Neurology, Department of Morphological Sciences, Faculty of Medicine, University of Santiago de Compostela, 15782 Santiago de Compostela, Spain; 2 Neurosciences Division, CIMA, University of Navarra, Pamplona, Spain; 3 Networking Research Center on Neurodegenerative Diseases (CIBERNED), Madrid, Spain

**Keywords:** Angiotensin, Dopamine, Basal ganglia, Parkinson, Intracrine, Neurodegeneration

## Abstract

We have previously obtained in rodents a considerable amount of data suggesting a major role for the brain renin–angiotensin system (RAS) in dopaminergic neuron degeneration and potentially in Parkinson’s disease. However, the presence of a local RAS has not been demonstrated in the monkey or the human substantia nigra compacta (SNc). The present study demonstrates the presence of major RAS components in dopaminergic neurons, astrocytes and microglia in both the monkey and the human SNc. Angiotensin type 1 and 2 and renin–prorenin receptors were located at the surface of dopaminergic neurons and glial cells, as expected for a tissular RAS. However, angiotensinogen and receptors for angiotensin and renin–prorenin were also observed at the cytoplasm and nuclear level, which suggests the presence of an intracrine or intracellular RAS in monkey and human SNc. Although astrocytes and microglia were labeled for angiotensin and prorenin receptors in the normal SNc, most glial cells appeared less immunoreactive than the dopaminergic neurons. However, our previous studies in rodent models of PD and studies in other animal models of brain diseases suggest that the RAS activity is significantly upregulated in glial cells in pathological conditions. The present results together with our previous findings in rodents suggest a major role for the nigral RAS in the normal functioning of the dopaminergic neurons, and in the progression of the dopaminergic degeneration.

## Introduction

The renin–angiotensin system (RAS) has long been considered as a circulating humoral system that regulates blood pressure and water homeostasis. Angiotensin II (AngII) is the most important effector peptide, and is formed by the sequential action of two enzymes, renin and angiotensin converting enzyme (ACE), on the precursor glycoprotein angiotensinogen. The actions of AngII are mediated by two main cell receptors: AngII type 1 and 2 (AT1 and AT2; Allen et al. 1998; Unger et al. 1996). AT_1_ receptors mediate most of the classical peripheral actions of AngII. However, AngII (via AT2 receptor) exerts actions directly opposed to those mediated by AT_1_ receptors thus antagonizing many of the effects of the latter (Chabrashvili et al. 2003; Jones et al. 2008). It is now generally accepted that in addition to the “classical” humoral RAS there exist local RAS in many tissues, including brain tissue (Ganong 1994; Re 2004), and that locally formed AngII regulates processes such as cell growth/apoptosis and inflammation (Ruiz-Ortega et al. 2001; Suzuki et al. 2003). Local AngII (via AT1 receptors) activates NADPH-dependent oxidases (Garrido and Griendling 2009; Touyz 2004), which are a major source of superoxide and are upregulated in major aging-related diseases such as hypertension, diabetes and atherosclerosis (Benigni et al. 2009; Griendling et al. 2000; Münzel and Keany 2001). However, activation of AT2 receptors inhibits NADPH-oxidase activation. Emerging aspects of the RAS, such as the identification of a specific receptor for renin and its precursor prorenin (PRR) (Nguyen 2011; Nguyen et al. 2002) and the identification of a functional intracellular/intracrine RAS in several types of cells, in addition to the “classical” humoral RAS and the local or tissue RAS (Baker et al. 2004; Re 2003), are contributing to a better understanding of the RAS and RAS-related diseases (Kumar et al. 2007, 2009).

The brain has an independent local RAS, which was initially associated with the central control of blood pressure. Over the last two decades, several components of the classical RAS have been identified in the brain (Cuadra et al. 2010; Phillips and de Oliveira 2008; Saavedra 2005; Wright and Harding 2011). Circulating AngII does not cross the brain–blood barrier, and the astroglia is considered as the main site of angiotensinogen synthesis in the brain (Milsted et al. 1990; Stornetta et al. 1988), although several authors have suggested that angiotensinogen is probably produced at lower levels in neurons (Kumar et al. 1988; Thomas et al. 1992). The existence of brain renin (Ganten et al. 1971) was initially controversial matter, probably due to the low expression levels of renin in comparison with the brain levels of AngII. However, the recent location of the new prorenin/renin receptor (PRR) in brain tissue has helped to clarify this issue (Nguyen et al. 2002).

More recent studies have involved the brain RAS in several brain disorders, including stroke (Li et al. 2005; Lou et al. 2004) and Alzheimer’s disease (Kehoe and Wilcock 2007; Mogi and Horiuchi 2009; Savaskan 2005). Over the last few years, we have carried out numerous experiments with rodents and have obtained a considerable amount of data suggesting a major role for the brain RAS in dopaminergic neuron degeneration and potentially in Parkinson’s disease (PD). We have observed all major components of the RAS system in dopaminergic neurons and glial cells of rat and mouse substantia nigra compacta (SNc), (Joglar et al. 2009; Rodriguez-Pallares et al. 2008). We have observed that in rodents RAS hyperactivation exacerbates NADPH-oxidase activity, oxidative stress and the microglial inflammatory response, which all contribute to the progression of dopaminergic neuron loss (Joglar et al. 2009; Lopez-Real et al. 2005; Muñoz et al. 2006; Rey et al. 2007; Rodriguez-Pallares et al. 2008). However, the presence of the major components of the RAS has not been demonstrated in primate or human SNc, which would provide further support for the involvement of brain RAS in PD. In the present study, we have demonstrated the location of the major components of the RAS in dopaminergic neurons and glial cells in the SNc of monkeys and humans, as well as the existence of an intracellular RAS in the SNc.

## Materials and methods

### Human postmortem and nonhuman primate tissue preparation

The human postmortem samples used in the present study were obtained from the Neurological Brain Bank of Navarra (Hospital of Navarra, Pamplona, Spain). Brains were dissected at autopsy from donors who had given informed consent in accordance with the Brain Donation Program of the Government of Navarra (Government directive 23/2001). The samples were obtained from four adult men (33.75 ± 6.4 years old) with no history or histological findings supporting any neurological disease. Following autopsy, brain slices including ventral mesencephalon were obtained, immediately frozen at −80°C and stored until processing. Postmortem times varied from 2.5 to 6 h.

Brain blocks including the mesencephalon were fixed by immersion in phosphate-buffered 4% paraformaldehyde for 24 h and cryoprotected for 48 h in a solution containing 20% of glycerin and 2% DMSO in 0.125 M phosphate buffer (PB), pH 7.4. Frozen coronal sections (40 µm thick) were then cut with a sliding microtome. Sections at different rostrocaudal levels of the SNc were processed by immunohistochemistry or immunofluorescence.

Nonhuman primate tissue was obtained from three adult male cynomolgus monkeys (*Macaca fascicularis*) (4.5–5 years old; body weight ranging from 3.8 to 4.5 kg). Animal handling was conducted in accordance with European Council Directive 86/609/EEC, and with the Society for Neuroscience Policy on the Use of Animals in Neuroscience Research. The experimental design was approved by the Ethical Committee for Animal Testing of the University of Navarra (ref: 019/2008) as well as by the Department of Health of the Government of Navarra (ref: NA-UNAV-04-08). Animals were anesthetized with an overdose of chloral hydrate and perfused transcardially with a fixative solution containing 4% paraformaldehyde and 0.1% glutaraldehyde in 0.125 M PB, pH 7.4. This very low amount of glutaraldehyde did not affect immunostaining and the brain sections can be used for additional studies with tracers that require a minimum amount of glutaraldehyde, and minimizes the number of monkeys used for research. Once perfusion was completed, the skull was opened, and the brain was removed and stored for 48 h in a cryoprotectant solution containing 20% of glycerin and 2% DMSO in 0.125 M PB, pH 7.4. Coronal tissue sections (40 µm thick) were then cut with a sliding microtome and collected in cryoprotectant solution. Sections at different rostrocaudal levels of the SNc were processed by immunohistochemistry or immunofluorescence.

### Histological processing: immunoperoxidase

A free-floating immunoperoxidase labeling method was used to evaluate the expression of angiotensinogen/angiotensin, AT1R, AT2R and PRR immunoreactivity in monkey and human SNc. Firstly, endogen peroxidase activity was inhibited in SNc sections with a solution of hydrogen peroxide (H_2_O_2_; 3%; Merck) diluted in potassium phosphate buffer saline (KPBS). Antigen retrieval was achieved in human SNc sections by incubation with 10 mM sodium citrate buffer (pH = 3.5) for 30 min at 37°C. Tissue sections were then pre-incubated in KPBS containing bovine serum albumin (BSA, 1%; Sigma), normal swine or horse serum (4%; Vector Laboratories) and Triton X-100 (0.05%; Sigma) for 60 min at room temperature (RT). Tissue sections were subsequently incubated with different polyclonal antibodies raised against major RAS components (Table 1): angiotensinogen (goat IgG; 1:200; AF3156, R&D Systems, RD; detects human angiotensinogen), angiotensin (Angiotensin N-10, goat IgG; 1:1,000; sc-7419, Santa Cruz Biotechnology, SC; detects human angiotensinogen, angiotensin I, II and III), AT1R (rabbit IgG; 1:100; sc-579, Santa Cruz Biotechnology), AT2R (rabbit IgG; 1:100; sc-9040, Santa Cruz Biotechnology) and PRR (rabbit IgG; 1:100, ab40790; Abcam) for 48 h at 4°C in a dilution of KPBS–BSA (1%) containing 2% normal swine or horse serum. Following primary antibody labeling, tissue sections were first incubated for 60 min at RT in the corresponding biotinylated secondary antibodies (biotinylated swine anti-rabbit IgG, 1:200, Dako; biotinylated horse anti-goat IgG, 1:200, Vector laboratories). Tissue sections were then incubated with the avidin–biotin-peroxidase complex (1:70; Vectastain Elite ABC kit; Vector Laboratories) for 60 min at RT. Staining for peroxidase was performed in KPBS with 0.05% 3-3′ diaminobenzidine tetrahydrochloride (Sigma) and 0.04% H_2_O_2_. Tissue sections were mounted on gelatin-coated slides and coverslipped using DPX (Panreac). Finally, sections were visualized with a Nikon Optiphot-2 microscope connected to a digital camera (Nikon DXM1200). No staining was observed in control sections in which primary antibodies were omitted from the incubation solution. We previously confirmed the specificity of the antibodies used in the present study by Western blot analysis with the corresponding peptide-preabsorbed antibody (Rodriguez-Perez et al. 2010; Valenzuela et al. 2010).

**Table 1:** Primary antibodies

Antibody specificity	Host species	Type (clone)	Isotype	Dilution	Manufacturer (cat. no.)
Angiotensin II type 1 receptor	Rabbit	Polyclonal	IgG	IP: 1:100	Santa cruz biotechnology (sc-579)
				IF: 1:50	
Angiotensin II type 2 receptor	Rabbit	Polyclonal	IgG	IP: 1:100	Santa cruz biotechnology (sc-9040)
				IF: 1:50	
Angiotensinogen	Goat	Polyclonal	IgG	IP: 1:200	R&D systems (AF3156)
				IF: 1:100	
Angiotensinogen, angiotensin I, II and III	Goat	Polyclonal	IgG	IP: 1:1,000	Santa cruz biotechnology (sc-7419)
				IF: 1:500	
Glial fibrillary acidic protein	Mouse	Monoclonal (GA5)	IgG1	IF: 1:500	Millipore (MAB360)
HLA-DR	Mouse	Monoclonal (169-1B5)	IgG2b	IF: 1:50	MP biomedicals (68549)
HLA-DR	Mouse	Monoclonal (LN-3)	IgG2b	IF: 1:50	Novocastra (NCL-LN3)
Renin/prorenin receptor	Rabbit	Polyclonal	IgG	IP: 1:100	Abcam (ab40790)
				IF: 1:50	
Tyrosine hydroxylase	Mouse	Monoclonal (TH-16)	IgG	IF: 1:5,000	Sigma (T2928)

*IP* immunoperoxidase labeling method, *IF* immunofluorescence labeling method

### Histological processing: double immunofluorescence labeling

Double immunofluorescence labeling was performed to identify the cells that expressed angiotensinogen/angiotensin, AT1R, AT2R and PRR in the human and monkey SNc. Angiotensinogen/angiotensin, AT1R, AT2R and PRR antibodies were combined with antibodies against tyrosine hydroxylase (TH; as a marker of dopaminergic neurons), glial fibrillary acidic protein (GFAP; as a marker of astrocytes), and human HLA class II-DR (as a marker of both resting and reactive microglia; Miles and Chou 1988; Verina et al. 2011). Neuromelanin granules detected by bright-field microscopy co-localized with TH in human SNc sections, and were also used as markers of dopaminergic neurons. Free-floating tissue sections containing SNc were pre-incubated in KPBS-1% BSA with 4% normal donkey serum (Sigma) and 0.05% Triton X-100 for 60 min at RT. Antigen retrieval was required for human SNc sections. Tissue sections were then incubated for 66–72 h at 4°C in primary antibodies (Table 1) raised against angiotensinogen (RD; 1:100), angiotensin (SC; 1:500), AT1R (1:50), AT2R (1:50), PRR (1:50), TH (1:5,000; mouse monoclonal; T2928, Sigma), GFAP (1:500; mouse monoclonal; MAB360, Millipore), HLA-DR (1:50; mouse monoclonal; 68549, MP Biomedicals) or HLA-DR (1:50; mouse monoclonal; NCL-LN3, Novocastra) diluted in KPBS-1% BSA with 2% normal donkey or rabbit serum. The immunoreaction was visualized with the fluorescent secondary antibodies: Alexa Fluor 568-conjugated donkey anti-rabbit IgG (1:200; Molecular Probes) or Alexa Fluor 488-conjugated donkey anti-mouse IgG (1:200; Molecular Probes) or Cy3-conjugated rabbit anti-goat IgG (1:200; Millipore). Finally, tissue sections were incubated for 30 min at RT with the DNA-binding dye Hoechst 33342 (3 × 10^−5^ M in KPBS), mounted on gelatin-coated slides and coverslipped with Immumount (Thermo-Shandon).

Tissue sections were visualized with a confocal laser-scanning microscope (TCS-SP2; Leica Microsystems Heidelberg GmbH, Mannheim, Germany). Confocal images were obtained by a sequential scan method and three different laser lines to avoid simultaneous excitation and possible overlap. Emission from the blue diode at 405 nm was detected in a spectral range of 422–457 nm and color-coded in blue. Emission from the argon laser at 488 nm was detected in a spectral range of 500–535 nm and color-coded in green. Finally, a spectral range of 581–625 nm was used to visualize the emission from the DPS diode at 561 nm, which was color-coded in red. Co-localization analysis was subsequently performed with the captured images in order to detect double-labeled cells. Series of confocal images were obtained every 0.7 μm in the *Z*-axis level by use of a sequential scan method. Analysis of the photographs at central cell levels revealed whether the labeling was located as a peripheral ring (suggesting membrane labeling) or throughout the cytoplasm. The presence of immunofluorescent labeling in neurons containing neuromelanin was analyzed using images obtained with the TCS-SP2 laser confocal microscope. To study co-localization of neuromelanin and immunofluorescence, emission from the DPS diode at 561 nm was detected in a spectral range of 581–625 nm and color-coded in green, and the bright-field illumination was acquired using an independent detection canal and color-coded in the gray scale.

## Results

**Fig. 1:**
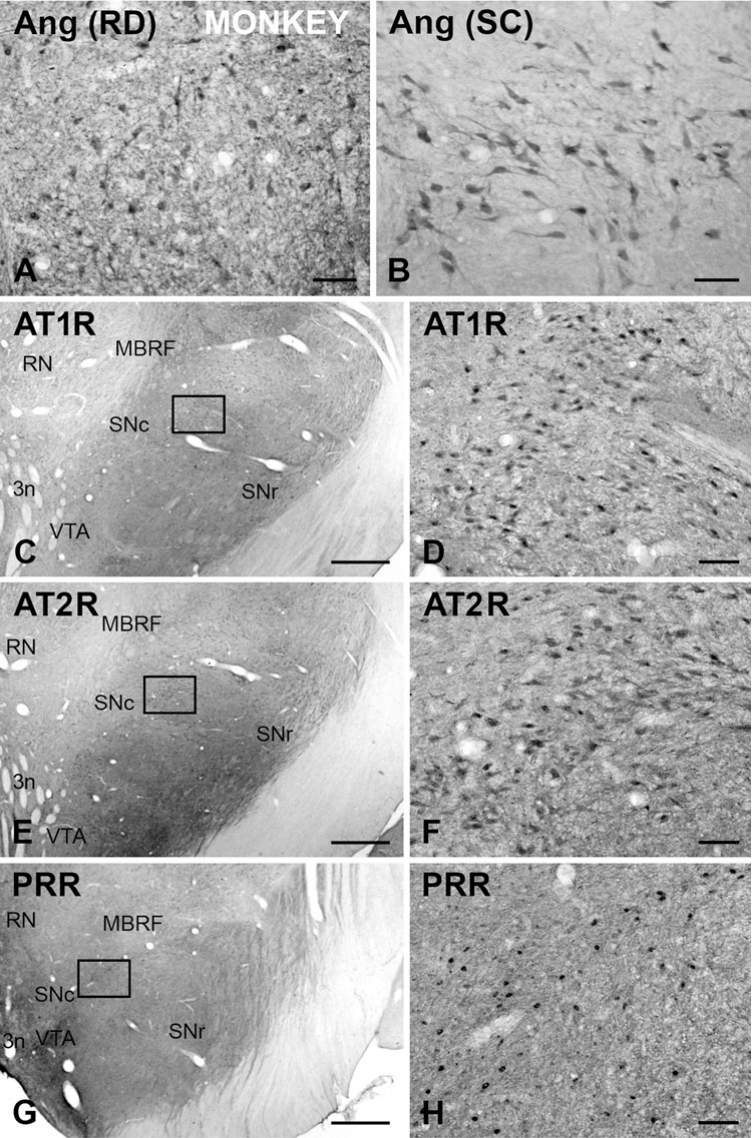
Coronal sections through the monkey ventral mesencephalon showing immunoperoxidase labeling for angiotensinogen/angiotensin (**a**, **b**), or AT1R (**c**, **d**), or AT2R (**e**, **f**), or PPR (**g**, **h**) show a large number of immunoreactive cells in the substantia nigra compacta. **a**, **b**, **d**, **f**, **h** show high magnification photographs of the *areas boxed* in **c**, **e**, **g**. *3n* third cranial nerve, *Ang* angiotensinogen/angiotensin, *AT1R* angiotensin II type 1 receptor, *AT2R* angiotensin II type 2 receptor, *MBRF* midbrain reticular formation, *PRR* prorenin/renin receptor, *RD* R&D systems, *RN* red nucleus, *SC* Santa Cruz Biotechnology, *SNc* substantia nigra pars compacta, *SNr* substantia nigra pars reticulata, *VTA* ventral tegmental area. *Scale bars* 100 µm (**a**, **b**, **d**, **f**, **h**); 1 mm (**c**, **e**, **g**)

**Fig. 2:**
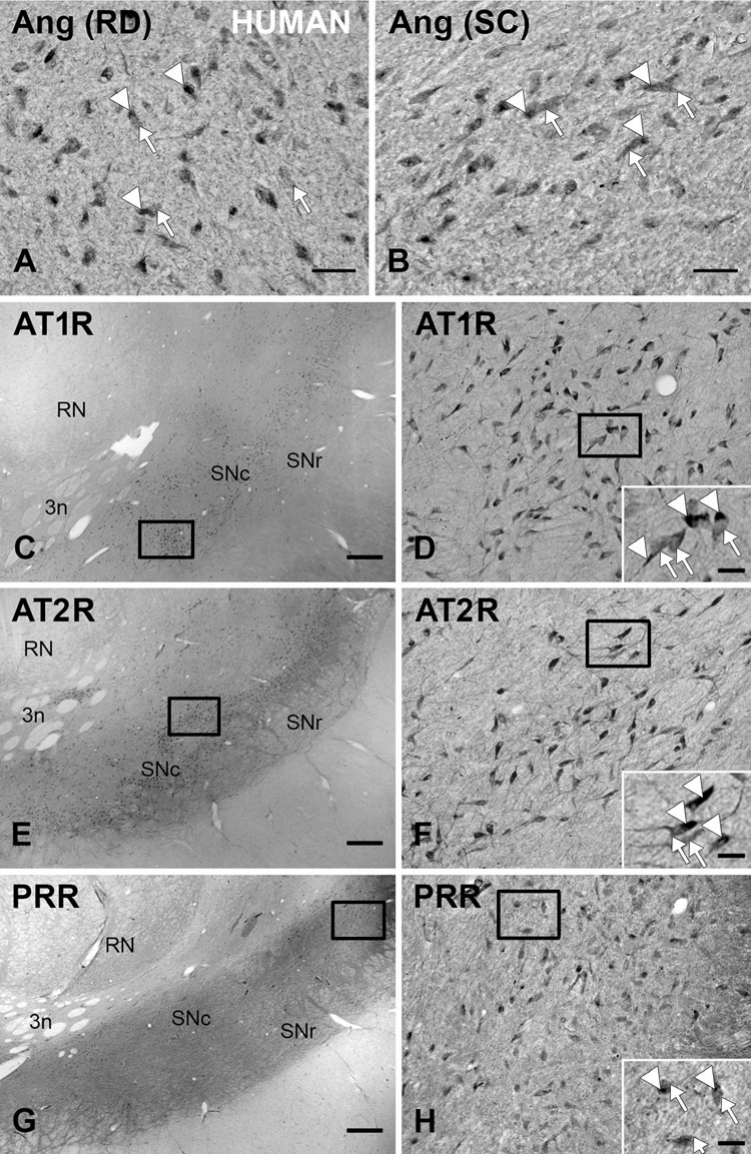
Coronal sections through the human ventral mesencephalon showing immunoperoxidase labeling for angiotensinogen/angiotensin (**a**, **b**), or AT1R (**c**, **d**), or AT2R (**e**, **f**), or PPR (**g**, **h**) show a large number of immunoreactive cells in the substantia nigra compacta. **a**, **b**, **d**, **f**, **h** show high magnification photographs of the *areas boxed* in **c**, **e**, **g**. Enlargements of the *areas boxed* in **d**, **f**, **h** show the presence of neuromelanin granules (*arrowheads*) together with AT1R, AT2R or PPR immunolabeling (*arrows*). *3n* third cranial nerve, *Ang* angiotensinogen/angiotensin, *AT1R* angiotensin II type 1 receptor, *AT2R* angiotensin II type 2 receptor, *PRR* prorenin/renin receptor, *RD* R&D systems, *RN* red nucleus, *SC* Santa Cruz Biotechnology, *SNc* substantia nigra pars compacta, *SNr* substantia nigra pars reticulata. *Scale bars* 100 µm (**a**, **b**, **d**, **f**, **h**); 1 mm (**c**, **e**, **g**); 50 µm (enlargements in **d**, **f**, **h**)

Single immunoperoxidase stains of sections from monkey and human ventral mesencephalon revealed intense immunoreactivity for angiotensinogen/angiotensin, AT1 and AT2 and PRR receptors in a large number of cells in the SNc, as well as in the ventral tegmental area (VTA) and other areas of the monkey and human ventral mesencephalon (Figs. 1, 2). The specificity of the labeling was confirmed using control sections in which the primary antibodies were omitted. The control sections did not exhibit immunoreactive labeling, and only neuronal profiles with neuromelanin granules were observed in the human SNc, thus revealing the location of the dopaminergic neurons.

**Fig. 3:**
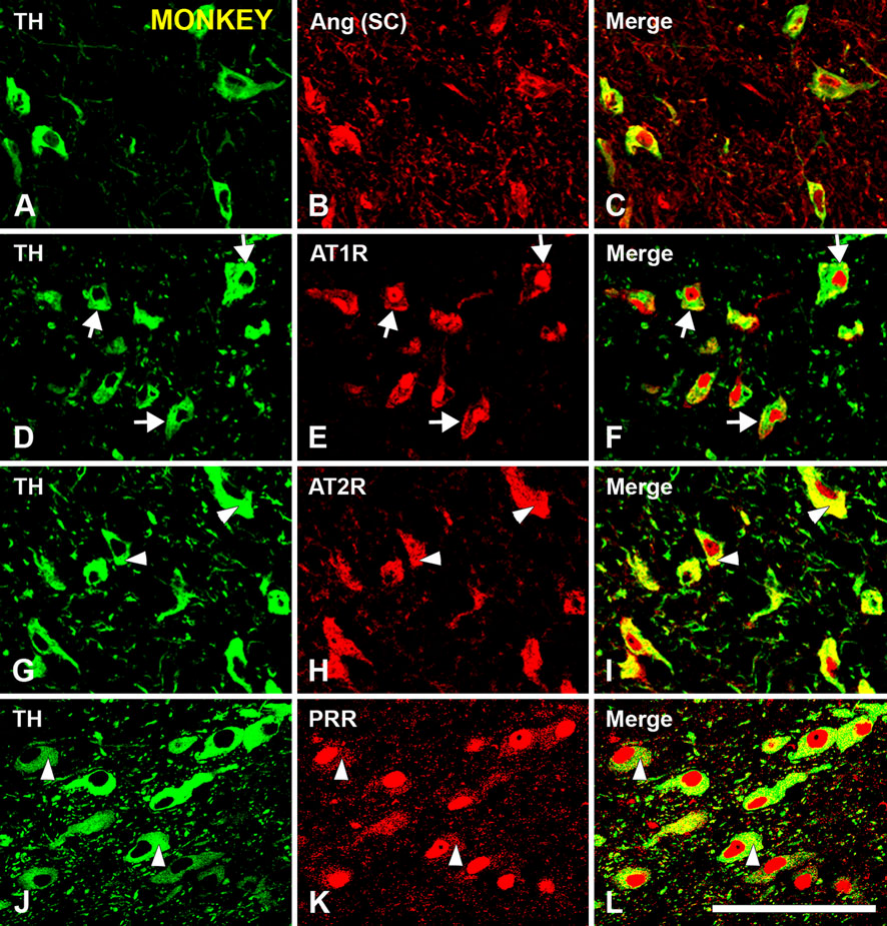
Double immunofluorescence for tyrosine hydroxilase (*green* dopaminergic neurons) and angiotensinogen/angiotensin (Ang; SC, Santa Cruz Biotechnology) or AT1R or AT2R or PRR (*red*) in the monkey substantia nigra compacta. Ang (**a**–**c**) and AT1R (**d**–**f**), AT2R (**g**–**i**) and PRR (**j**–**l**) show co-localization (*yellow*) with the dopaminergic marker TH. Labeling is apparently located at cell surface (*arrows* in **d**–**f**), neuron nucleus, and cytoplasmic (*arrowheads* in **g**–**l**) levels. The cytoplasmic labeling for PRR was weaker than for AT1R and AT2R, but clearly visible at high magnification (*arrowheads* in **j**–**l**). *Ang* angiotensinogen/angiotensin, *AT1R* angiotensin II type 1 receptor, *AT2R* angiotensin II type 2 receptor, *PRR* prorenin/renin receptor, *SC* Santa Cruz Biotechnology, *RD* R&D Systems, *TH* tyrosine hydroxylase *Scale bar* 100 µm

**Fig. 4:**
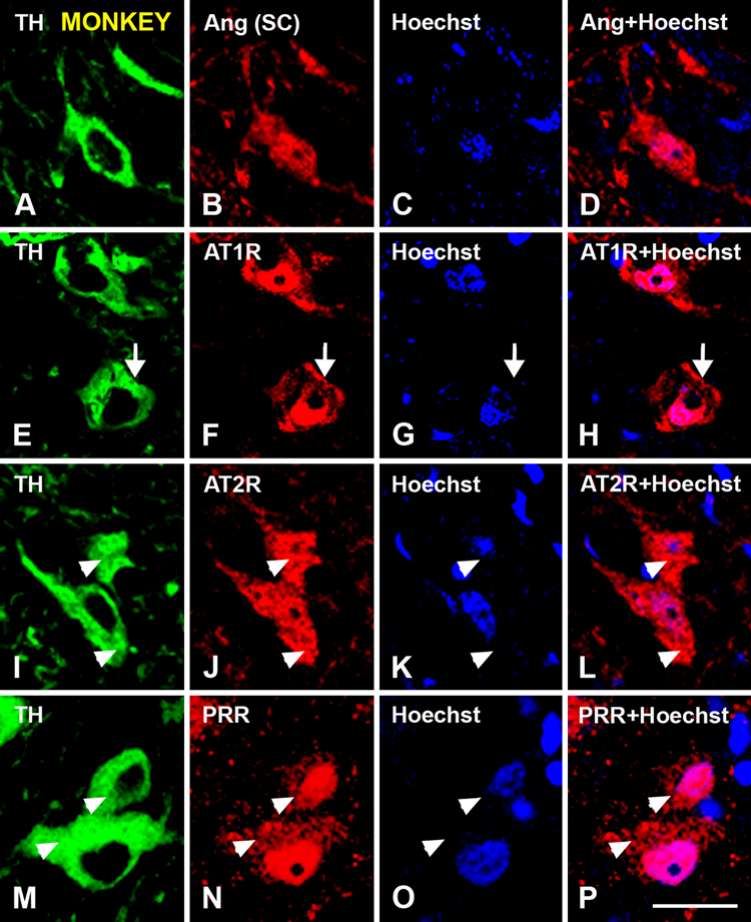
Triple immunofluorescent labeling showing dopaminergic neurons at high magnification in the monkey substantia nigra compacta (TH-ir; **a**, **e**, **i**, **m**; *green*), and double-labeled for the nuclear marker Hoechst 33342 (*blue*) and (*red*) angiotensinogen/angiotensin (**a**–**d**), AT1R (**e**–**h**), AT2R (**i**–**l**) and PRR (**m**–**p**). Labeling is apparently located at cell surface (*arrows* in **e**–**h**), cytoplasmic (*arrowheads* in **i**–**p**) and nuclear levels. AT1R labeling is most intense at the cell surface and in the nucleus (**e**–**h**), AT2R labeling in the cytoplasm (**i**–**l**) and PRR labeling at the nuclear level (**m**–**p**). The weak cytoplasmic labeling for PRR is shown by *arrowheads* in **m**–**p**. *Ang* angiotensinogen/angiotensin, *AT1R* angiotensin II type 1 receptor, *AT2R* angiotensin II type 2 receptor, *PRR* prorenin/renin receptor, *SC* Santa Cruz Biotechnology, *TH* tyrosine hydroxylase. *Scale bar* 25 µm

**Fig. 5:**
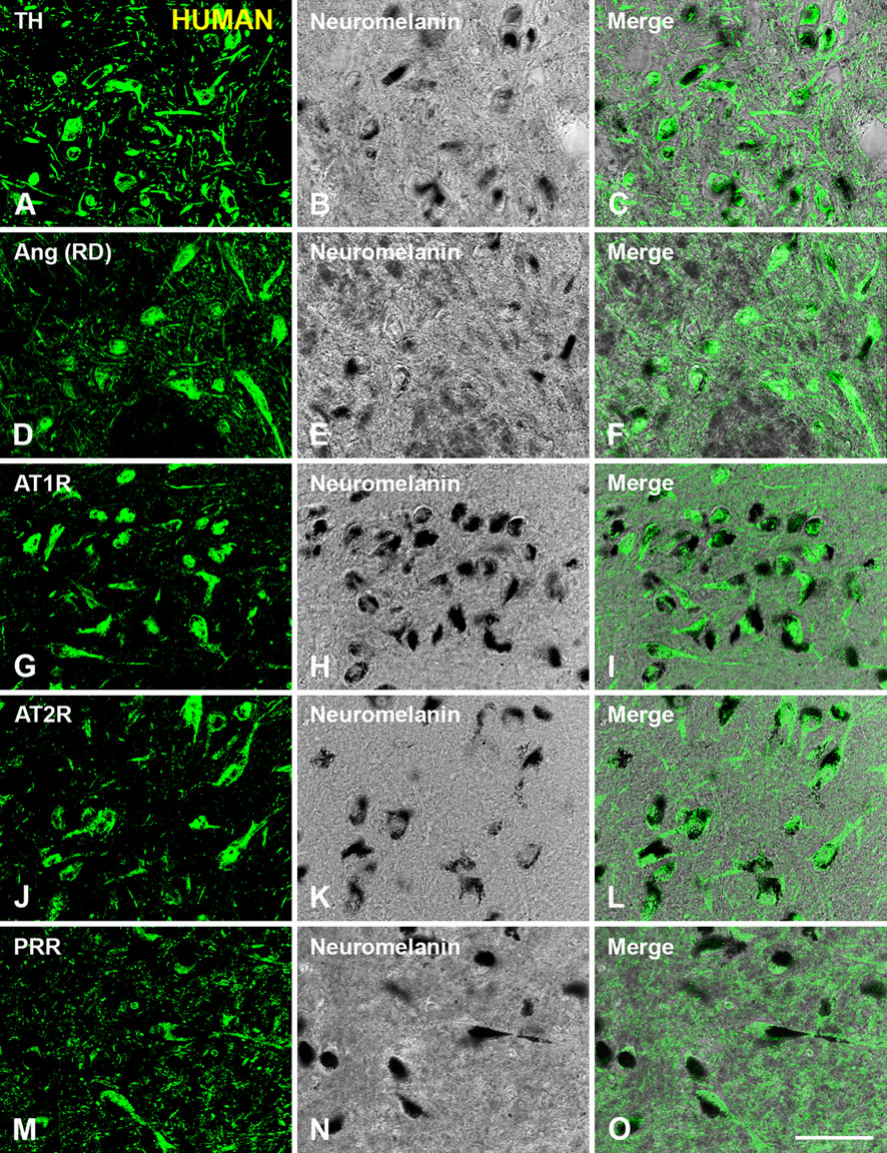
Immunofluorescent labeling of dopaminergic neurons in the human substantia nigra compacta were identified either with the dopaminergic marker tyrosine hydroxylase or clearly visible neuromelanin granules, which co-localized in dopaminergic neurons as shown in **a**–**c**. Angiotensinogen/angiotensin (**d**–**f**), AT1R (**g**–**i**), AT2R (**j**–**l**), and PRR (**m**–**o**) were located in all dopaminergic neurons containing neuromelanin granules. *Ang* angiotensinogen/angiotensin, *AT1R* angiotensin II type 1 receptor, *AT2R* angiotensin II type 2 receptor, *PRR* prorenin/renin receptor, *RD* R&D Systems, *TH* tyrosine hydroxylase. *Scale bar* 100 µm

Double immunofluorescence and confocal microscopy were used to identify the SNc cells that expressed angiotensinogen/angiotensin, AT1, AT2 receptors and PPR. Dopaminergic neurons were identified by their TH-immunoreactivity in monkey sections (Figs. 3, 4) and TH-immunoreactivity or the presence neuromelanin granules in human sections (Fig. 5). Co-localization of TH-immunofluorescence and neuromelanin granules was confirmed in human SNc neurons by confocal microscopy (Fig. 5a–c). Ultrastructural analysis is required to confirm unequivocally that labeling is located in the cytoplasm or membrane. However, series of confocal images were obtained every 0.7 μm in the *Z*-axis level by use of a sequential scan method. Analysis of the photographs at central cell levels revealed whether the labeling was located as a peripheral ring (suggesting membrane labeling) or throughout the cytoplasm. In the monkey, dopaminergic neurons were intensely immunopositive for all major RAS components studied. Angiotensinogen/angiotensin was located in both the cytoplasm and neuronal nucleus (Figs. 3a–c, 4a–c). Labeling for AT1 receptors was intense at the periphery of the neurons (Figs 3d–f, 4d–f), suggesting the presence of receptors at the cell surface. However, AT1R labeling was also intense within the neurons, particularly at nuclear level, as revealed by the use of Hoechst stain as a nuclear marker (Fig. 4d–f). AT2 receptor labeling appeared also located at the cell surface and the neuronal nucleus, and appeared particularly intense in the cytoplasm (Figs. 3g–i, 4g–i). PPR labeling was particularly intense in the neuronal nucleus (Figs. 3j–l, 4j–l), although weaker labeling was distinguished at the cell surface and in the cytoplasm. Intense labeling for angiotensinogen/angiotensin, AT1R, AT2R and PRR was also observed in human dopaminergic neurons. Labeling for the different receptors was apparently located at the cell surface, and in the cytoplasm and nucleus as described above for monkeys (Fig. 5).

**Fig. 6:**
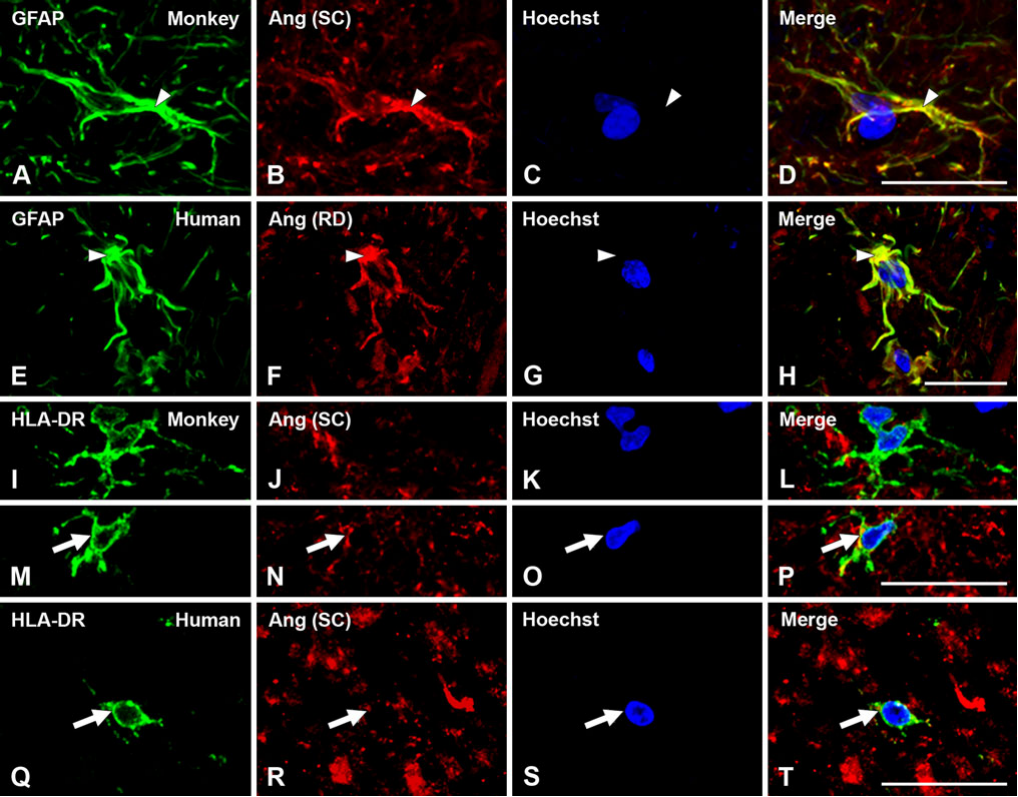
Triple immunofluorescent labeling for the astroglial marker GFAP (**a**–**h**; *green*) or the microglial marker HLA-DR (**i**–**t**; *green*), the nuclear marker Hoechst 33342 (*blue*), and angiotensinogen/angiotensin (Ang; *red*) in monkey and human substantia nigra compacta. In astrocytes, labeling is mainly located at the cytoplasmic level. In microglial cells of normal substantia nigra, labeling for angiotensinogen/angiotensin was weak and particularly located at the cell surface (*arrows*). *Ang* angiotensinogen/angiotensin, *GFAP* glial fibrillary acidic protein, *HLA-DR* human leukocyte antigen DR, *SC* Santa Cruz Biotechnology, *RD* R&D Systems. *Scale bar* 20 µm

**Fig. 7:**
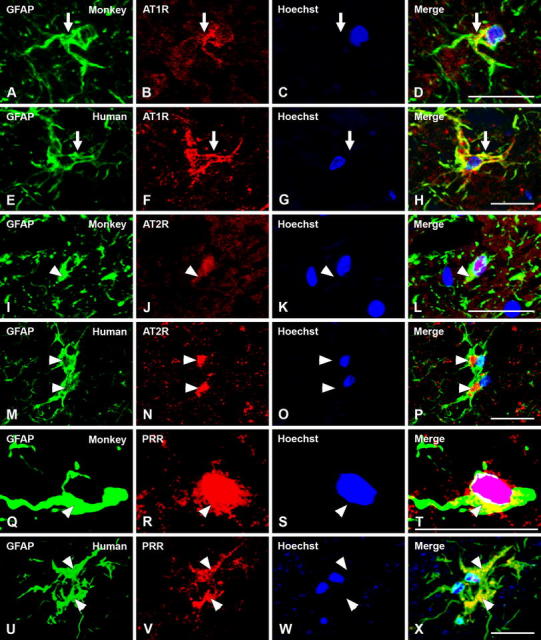
Triple immunofluorescent labeling for the astroglial marker GFAP (*green*), the nuclear marker Hoechst 33342 (*blue*), and AT1R, AT2R or PRR (*red*) in monkey and human substantia nigra compacta. Labeling for AT1R appeared mostly located at the cell surface (*arrows* in **a**–**h**), and AT2R and PRR labeling at the cytoplasm and nuclear levels (*arrowheads* in **i**–**x**). *AT1R* angiotensin II type 1 receptor, *AT2R* angiotensin II type 2 receptor, *GFAP* glial fibrillary acidic protein, *PRR* prorenin/renin receptor. *Scale bar* 20 µm

**Fig. 8:**
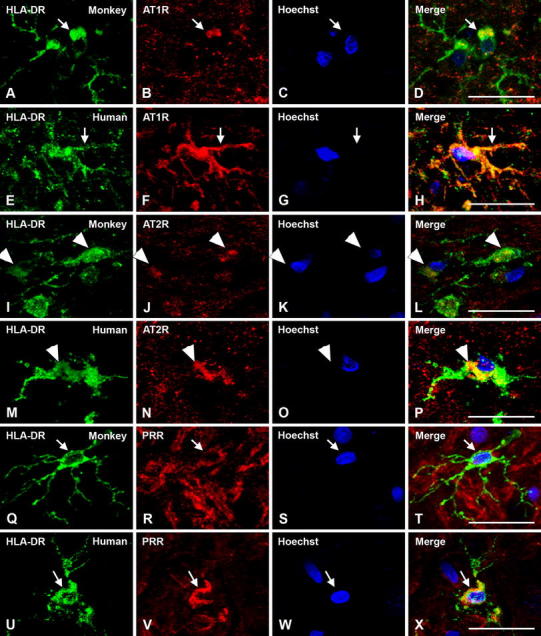
Triple immunofluorescent labeling for the microglial marker HLA-DR (*green*), the nuclear marker Hoechst 33342 (*blue*), and AT1R, AT2R or PRR receptors (*red*) in monkey and human substantia nigra compacta. Labeling for AT1R is apparent at the cell surface, cytoplasm and nuclear levels (**a**–**h**, *arrows*); labeling for AT2R is apparent mainly at the cytoplasmic level (**i**–**p**, *arrowheads*), and PRR mainly at the cell surface level (**q**–**x**, *arrows*). *AT1R* angiotensin II type 1 receptor, *AT2R* angiotensin II type 2 receptor, *HLA-DR* human leukocyte antigen DR, *PRR* prorenin/renin receptor. *Scale bar* 20 µm

All major RAS components studied (angiotensinogen/angiotensin, AT1R, AT2R and PRR) were also located in glial cells in the SNc of monkeys and humans (Figs. 6, 7, 8). In monkey and human SNc, astrocytes were identified by GFAP immunofluorescence, and exhibited intense labeling for angiotensinogen/angiotensin located in the cytoplasm, without a clear labeling at the nuclear level (Fig. 6a–h). AT1 receptors were mainly located at the cell surface and in the cytoplasm, although weak labeling for AT1R was occasionally observed at the nuclear level (Fig. 7a–h). AT2 receptor labeling was also observed in monkey and human astrocytes, particularly concentrated at nuclear and cytoplasmatic levels (Fig. 7i–p). Most astrocytes identified with GFAP did not show clear labeling for PRR in monkey and human SNc. However, a few astrocytes with unequivocal labeling for PRR were also observed revealing PRR in nuclear (Fig. 7q–t) and cytoplasmic (Fig. 7u–x) location.

Microglial cells were identified in human and monkey SNc sections by their HLA-DR immunoreactivity. In microglial cells of normal substantia nigra labeling for angiotensinogen/angiotensin was weak and mainly located at the cell surface (Fig. 6i–t). Immunoreactivity for AT1 receptors was observed at the cell surface and in the cytoplasm, and was also co-localized with the nuclear marker Hoechst (Fig. 8a–h). Human and monkey microglial cells were also immunoreactive for AT2 receptors, which was more intense in the cytoplasm at the perinuclear level (Fig. 8i–p). Immunoreactivity for PRR was also observed in monkey and human microglia (Fig. 8q–x). In most microglial cells PRR labeling appeared to be concentrated at the cell surface, although perinuclear or weak nuclear labeling was observed in some cells.

## Discussion

In a series of recent studies (Joglar et al. 2009; Rodriguez-Pallares et al. 2008; Valenzuela et al. 2010), we demonstrated the presence of angiotensin receptors in nigral dopaminergic neurons and glial cells in rodents, as well as in rat primary mesencephalic cultures (Joglar et al. 2009; Rodriguez-Pallares et al. 2004, 2008). The present study shows the location of major components of the RAS in dopaminergic neurons, astrocytes and microglial cells of the monkey and human substantia nigra. AT1R and AT2R and PRR were located at the cell surface, as may be expected for a local or tissular RAS. However, angiotensinogen and angiotensin and PRR receptors were also observed at the cytoplasmic or nuclear level, which suggests the presence of an intracrine or intracellular RAS in monkey and human SNc. Several transmembrane receptors are known to accumulate in nuclei, particularly in nuclear membranes. Cells such as cardiomyocytes possess AngII receptors that couple to nuclear signaling pathways and regulate transcription. The observed intracellular location supports the possibility of an intracellular function for AngII, in addition to the effects induced by activation of cell surface AT1 and AT2 receptors. Extracellular AngII may act intracellularly by binding to AT1 receptors, which are subsequently internalized, or AngII may be synthesized within the cell. AT1R-dependent internalization of AngII has been described in a number of different cell types (Chen et al. 2000; Eggena et al. 1996; Lu et al. 1998). However, the demonstration of an intracellular location for PRR receptors in DA neurons suggests that some AngII may be formed intraneuronally, as previously suggested for heart cells (Baker et al. 2004). Intracellular AngII has been suggested to induce transcription of angiotensinogen and renin in response to binding to nuclear AT1 receptors in some cell types (Eggena et al. 1996). Therefore, our observation of intracellular PRR receptors supports the existence of an intracellular/intracrine RAS in the brain, and in the SNc in particular, as previously been suggested for other cell types (Lavoie et al. 2004; Re 2003).

Several studies have reported the presence of RAS components in the human basal ganglia (Allen et al. 1992; Quinlan and Phillips 1981). Early autoradiographic studies reported AT1 receptors in dopaminergic neurons, both in cell bodies in the SNc and in their terminal fields in the striatum of different mammals, including humans (Allen et al. 1992, 1998; Lenkei et al. 1997; Simonnet et al. 1981), and suggested that the density of AT1 receptors may be higher in human striatum and SNc than in rats and other mammals (Allen et al., 1992, 1998). However, the existence of brain renin (and therefore a local brain RAS) was initially controversial, largely because of low levels of renin expression, which were below the detection threshold of some standard assays. However, Immunoreactivity for renin has also been reported in other studies in neurons and glial cells in several areas of the rodent (Dzau et al. 1982; Fuxe et al. 1980) and human (Slater et al. 1980) brains. The existence of a local brain RAS was also questioned because brain levels of AngII may be much higher than the levels of renin detected. This may be now explained by the recent location of PRR receptors in the brain. High levels of PRR mRNA expression were initially observed in brain tissue homogenates (Nguyen et al. 2002), and we have observed abundant PRR receptors in neurons and glial cells in the monkey and human brain. PRR activates a catalytic activity similar to that of renin by binding of prorenin (previously considered inactive precursor of renin), and the prorenin to renin ratios are 5–10 times higher (and even up to 20–200 times higher in pathological conditions; Luetscher et al. 1985).

In the present study, intense immunoreactivity against the RAS components under study was observed in the monkey and human dopaminergic neurons. RAS appears to be involved in several functions in the normal brain. However, the interaction between AngII and dopamine is particularly interesting. An important interaction between dopamine and AngII receptors in peripheral tissues has been demonstrated in several recent studies, particularly with regard to the regulation of renal sodium excretion and cardiovascular function (Gildea 2009; Khan et al. 2008; Zeng et al. 2006). Recent evidence suggests that dopamine and AngII systems directly counterregulate each other in renal cells (Gildea 2009) and that abnormal counterregulatory interactions between dopamine and AngII play a major role in degenerative changes and hypertension (Li et al. 2008). In a recent study (Villar-Cheda et al. 2010), we have shown similar functional interactions and counterregulatory mechanisms in the striatum and SNc of rodents, and the present data suggest that similar interactions and counterregulatory mechanisms take place in humans. However, the present study not only shows that the tissular RAS is well developed in the SNc, but also that there is an important intracellular RAS in the dopaminergic neurons. The role of the intraneuronal RAS and its interactions with the tissular RAS are unknown, and are currently under study in our laboratory.

Although SNc astrocytes and microglial cells were labeled for angiotensin or prorenin receptors, most glial cells appeared less intensely immunoreactive than the dopaminergic neurons. However, it is important to note that we studied the SNc of normal human and monkey brains. Nonetheless, our previous studies in the SNc of rodent models of PD and studies in other animal models of brain diseases (Lanz et al. 2010; Lou et al. 2004; Stegbauer et al. 2009) suggest that the RAS activity is significantly upregulated in glial cells in lesioned brains. In rats lesioned with the dopaminergic neurotoxin 6-OHDA, we have observed a significant increase in the expression of AT1 and AT2 receptors in the SNc, apparently due to glial upregulation since the dopaminergic neurons had been eliminated by the toxin (Villar-Cheda et al. 2010). This issue was also confirmed in cultures of microglial cells, as activated microglial cells showed more intense nuclear labeling for AT1R, AT2R and PRR than that observed in the normal SNc in the present study (Joglar et al. 2009; Rodriguez-Pallares et al. 2008; Valenzuela et al. 2010). Together our studies suggest that RAS hyperactivity enhances microglial derived oxidative stress and neuroinflammation, which play a major role in degeneration of dopaminergic neurons and possibly the progression of PD. We have shown that AngII, via AT1 receptors, exacerbates dopaminergic cell death and that a RAS-induced increase in the glial response plays a major role (Joglar et al. 2009; Lopez-Real et al. 2005; Muñoz et al. 2006; Rey et al. 2007; Rodriguez-Pallares et al. 2008). This is consistent with recent findings showing that neuroinflammation and oxidative stress play a pivotal role at least in the progression of PD, and also with numerous studies showing that the local RAS plays a key role in the initiation and perpetuation of inflammation and oxidative damage in different tissues (Ruiz-Ortega et al. 2001; Suzuki et al. 2003; Zalba et al. 2001). However, it is not yet known whether the pro-oxidative and pro-inflammatory effects of the tissular RAS is completed or enhanced by the intracellular RAS, or whether the intracellular RAS plays a different role, or whether the intracellular RAS has different functions in neurons and glial cells.

The mechanisms responsible for PD have not been totally clarified (González-Hernández et al. 2010; Smith and Villalba 2008). However, some other data also suggest the potential involvement of RAS in PD. A marked reduction in AT1 receptors has been observed in the striatum of PD patients in comparison with normal brains, and attributed to the loss of dopaminergic terminals in PD (Allen et al. 1992, 1998). However, the decrease in AT1 receptor expression was possibly more closely related to the L-dopa treatment that the PD patients had received (Villar-Cheda et al. 2010), in accordance with our experimental data from rodents. Furthermore, increased ACE activity in the cerebrospinal fluid of patients with PD has been reported (Konings et al. 1994), as well as an association between genetic polymorphism of the ACE gene and PD (Lin et al. 2002). Finally, a major role of the brain RAS in the dopaminergic system function and dopaminergic degeneration is also supported by several recent studies in which we have shown hyperactivation of the nigral RAS in several animal models with increased vulnerability of dopaminergic neurons to degeneration (i.e. increased risk for PD). Higher nigral RAS activity has been observed in aged male rats than in young male rats (Villar-Cheda et al. 2012), in menopausal rats (either surgical or natural menopause) than in female rats with estrogen (Rodriguez-Perez et al. 2012), and in young male rats in comparison with young female rats (Rodriguez-Perez et al. 2011, 2012). We have confirmed that in aged male rats and menopausal females, aging and lack of estrogen enhances the dopaminergic cell death induced by dopaminergic neurotoxins, and that an increased nigral RAS activity is involved; we observed increased AT1 receptor expression, increased activation of the NADPH-oxidase complex and increased levels of the pro-inflammatory cytokines, which indicate a pro-oxidative, pro-inflammatory state in the substantia nigra. Increased RAS activity and dopaminergic vulnerability were reduced by treatment with the AT1R antagonists such as candesartan.

In conclusion, the present study reveals the presence of a local/tissue RAS and an intracellular RAS in the SNc of monkeys and humans, which suggests a major role for the nigral RAS in the normal function of the dopaminergic neurons. Our previous findings in mouse and rat models of PD suggest a major role for the nigral RAS in progression of the dopaminergic degeneration, and the present results confirm the presence of a well developed nigral RAS in monkeys and humans. A better comprehension of the role of the RAS in the SNc, and the subsequent RAS manipulation may lead to an effective neuroprotective strategy against PD as previously observed in cardiovascular and renal diseases.

## Abbreviations

3n: Third cranial nerve
ACE: Angiotensin converting enzyme
AngII: Angiotensin II
Ang (RD): Angiotensinogen (R&D Systems)
Ang (SC): Angiotensinogen (Santa Cruz Biotechnology)
AT1R: Angiotensin II type 1 receptor
AT2R: Angiotensin II type 2 receptor
BSA: Bovine serum albumin
GFAP: Glial fibrillary acidic protein
HLA-DR: Human leukocyte antigen DR
KPBS: Potassium phosphate buffer saline
MBRF: Midbrain reticular formation
PB: Phosphate buffer
PD: Parkinson’s disease
PRR: Prorenin/renin receptor
RAS: Renin–angiotensin system
RN: Red nucleus
RT: Room temperature
SNc: Substantia nigra compacta
SNr: Substantia nigra pars reticulata
TH: Tyrosine hydroxylase
VTA: Ventral tegmental area
